# Body mass index and comorbidity are associated with postoperative renal function after nephrectomy

**DOI:** 10.1590/S1677-5538.IBJU.2014.0383

**Published:** 2015

**Authors:** Lael Reinstatler, Zachary Klaassen, Brittani Barrett, Martha K. Terris, Kelvin A. Moses

**Affiliations:** 1 Department of Surgery, Section of Urology, Medical College of Georgia-Georgia Regents University, Augusta, GA

**Keywords:** Body Mass Index, Comorbidity, Nephrectomy

## Abstract

**Purpose:**

To explore the association of body mass index (BMI) and comorbidity with renal function after nephrectomy.

**Materials and Methods:**

We retrospectively analyzed 263 patients submitted to partial or radical nephrectomy from 2000-2013. Variables assessed included BMI, Charlson Comorbidity Index (CCI), race, tobacco use, tumor histology, surgical approach, Fuhrman nuclear grade, and tumor (T) classification. Glomerular filtration rate (GFR) was estimated using the Cockroft-Gault equation, adjusted for gender. Logistic regression was performed and included all interaction terms.

**Results:**

Median follow-up was 19.6 months (IQR 5.2, 53.7). Median preoperative GFR was 86.2mL/min/1.73m^2^ and median postoperative GFR was 68.4mL/min/1.73m^2^. BMI (OR 1.07, 95%CI 1.02-1.11), CCI (OR 1.19, 95%CI 1.04-1.37), and radical nephrectomy (OR 3.09, 95%CI 1.51-6.33) were significantly associated with a decline in renal function of ≥25%.

**Conclusion:**

BMI and CCI are associated with postoperative decline in renal function after nephrectomy. Additionally, radical nephrectomy is significantly associated with decreasing renal function compared to partial nephrectomy. These findings highlight the importance of assessing patient comorbidity in the decision making process for patients presenting with a renal mass.

## INTRODUCTION

Nephrectomy has long been established as the primary treatment for renal cell carcinoma (RCC) (1). With the advent of nephron sparing surgery (NSS), several studies have documented its superior performance vs. radical nephrectomy in regard to post-operative renal function (2–7). In addition, clinical features such as male gender, increasing tumor size, higher tumor complexity, and hilar clamping (vs. artery alone) have been reported to influence post-operative renal function (8, 9).

Diabetes and hypertension are well established risk factors for chronic kidney disease (CKD), and these disease processes have demonstrated associations with poorer post-operative renal function in patients submitted to nephrectomy (10). With the increasing incidence of renal cell carcinoma (RCC) (11) and therefore the inevitable increase in surgical procedures, it is important for the urologist to understand the impact of physiologic factors, in addition to clinical ones, on patient prognosis.

There is conflicting data regarding the association of body mass index (BMI) with renal function. Some studies find no association of BMI with a change in glomerular filtration rate (GFR), (12, 13) while others have found BMI to be an independent predictor of the development of CKD after partial nephrectomy (14, 15). It has also been proposed that BMI≤25 may be protective, as these patients may be more likely to have benign renal pathology (16).

Additional information is needed to elucidate a clearer picture of physiologic and clinical predictors of renal function after nephrectomy. Therefore, the objective of the current study was to evaluate the impact of BMI and patient comorbidity assessed by the Charlson Comorbidity Index (CCI) on renal function after nephrectomy for RCC.

## MATERIALS AND METHODS

After obtaining institutional review board approval, we constructed a retrospective database of patients at Georgia Regents University Medical Center who received either partial or radical nephrectomy for a renal mass between January 1, 2000 and April 30, 2013. After excluding patients who had surgery for extrarenal masses (i.e. retroperitoneal or urothelial in origin), a total of 263 patients were included in the final database.

Patient information for the following covariates was collected: BMI, CCI, race, age, follow-up time, tobacco use, primary tumor histology, American Joint Committee on Cancer (AJCC) 2007 (Tumor), N(ode), and M(etastasis) classification, and Fuhrman nuclear grade. Race was categorized as African-American or other. GFR was estimated using the Cockroft-Gault equation, adjusted for gender. Preoperative GFR represents baseline renal function at the preoperative visit within one month of the surgery date. Follow-up GFR was calculated based on clinical parameters at the most recent clinic visit. Tobacco use was classified as current/past user or never user. Follow-up time was bounded by our censoring date of April 30, 2013. Data on age was collected but it was later discarded as an individual variable and instead used in an age-adjusted CCI, as previously described (17). Data on gender was also collected but withheld from the analysis as the formula for GFR accounts for gender.

The outcome, decline in renal function, was assessed in two separate models. In the first model, a decline in renal function was defined as a postoperative decrease in GFR≥25%. In the second model, a decline in renal function was defined as postoperative GFR≤60mL/min/1.73m^2^. Since moderate to severe renal disease is a component of CCI, we adjusted this variable to remove potential confounding of the predictor with the outcome. For patients with moderate to severe renal disease, as defined by preoperative GFR≤60mL/min/1.73m^2^, 2 points were removed from their total CCI.

Median values and inter-quartile ranges were generated for all continuous variables. Frequencies and proportions were generated for categorical ones. Covariates assessed in the logistic regression model included BMI, age-adjusted CCI, race (other vs. African-American), tobacco use, tumor histology, partial vs. radical nephrectomy, Fuhrman grade, and T Classification. N and M classification was removed from the regression analysis as the small amount of data available in these categories created model instability.

Statistical analysis was performed using SAS 9.3 (SAS Institute, Cary, NC). All tests were two-sided with a significance level set at p<0.05. Categorical variables were assessed using the Chi-square test and continuous variables were assessed using the t-test. Logistic regression was performed using backwards selection to identify the best fit model and included all interaction terms.

## RESULTS

Patient demographic information is summarized in Table-1. The median follow-up time was 19.6 months and the mean follow-up time was 34.9 months. Median BMI was 28.3 (IQR 24.4, 32.6) and median CCI was 3 (IQR 1, 5). Median preoperative GFR was 86.2mL/min/1.73m^2^ (IQR 56.5, 114.3) and median postoperative GFR was 68.4mL/min/1.73m^2^ (IQR 43.9, 95.8). The majority of patients were male (57%) and used tobacco (54%). African-American patients represented 38.4% of the study population.

**Table 1:** Patient Demographics and Tumor Characteristics.

Variable		Median (25^th^, 75^th^%)
Age (years)		57.8 (49.6, 66.6)
BMI (kg/m^2^)		28.3 (24.4, 32.6)
CCI		3 (1, 5)
Preoperative Cr (mg/dL)		1.03 (0.8, 1.4)
Preoperative GFR (mL/min/1.73m^2^)		86.2 (56.5, 114.3)
Tumor Size (cm)		4.5 (2.7, 8.3)
Time to follow-up (mos)		19.6 (5.2, 53.7)
Cr at follow-up (mg/dL)		1.3 (1.0, 1.7)
GFR at follow-up (mL/min/1.73m^2^)		68.4 (43.9, 95.8)
		N (%)
Gender		
	Male	150 (57.0%)
	Female	113 (43.0%)
Race		
	African American	101 (38.4%)
	Other	162 (61.6%)
**Tobacco Use**		142 (54.0%)
**Tumor Site**		
	Left	135 (51.3%)
	Right	125 (47.5%)
	Bilateral	3 (1.2%)
**Surgery Type**		
	Partial	85 (32.3%)
	Radical	178 (67.7%)
**Tumor Histology**		
	Clear Cell	148 (56.3%)
	Papillary	53 (20.2%)
	Chromophobe	9 (3.4%)
	Benign	19 (7.2%)
Other		34 (12.9%)
**Fuhrman Grade**		
	G1	22 (9.5%)
	G2	105 (45.2%)
	G3	76 (32.8%)
	G4	29 (12.5%)
**T Classification**		
	pT1a	99 (40.9%)
	pT1b	55 (22.7%)
	pT2a	18 (7.4%)
	pT2b	15 (6.2%)
	pT3a	28 (11.6%)
	pT3b	21 (8.7%)
	pT3c	1 (0.4%)
	pT4	5 (2.1%)
**N Classification**		
	pNx	198 (81.5%)
	pN0	34 (14.0%)
	pN1	11 (4.5%)
**M Classification**		
	pMx	223 (91.8%)
	pM1	20 (8.2%)
**GFR Decrease ≥25%**		
	No	157 (61.3%)
	Yes	99 (38.7%)

**BMI** = body mass index; **CCI** = Charlson Comorbidity Index; **GFR** = glomerular filtration rate

The majority of patients underwent radical nephrectomy (67.7%: 20 of these were cytoreductive nephrectomy) while 32.3% had NSS. The histological type of renal masses was divided into clear cell RCC (56.3%), papillary RCC (20.2%), other (12.9%), benign (7.2%), and chromophobe RCC (3.4%). The most common grade of malignant tumors was Fuhrman nuclear grade G2 (45.2%), followed by G3 (32.8%).

**Figure 1:**
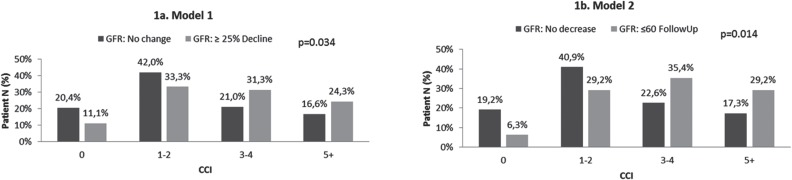
Association of CCI with Postoperative GFR.

Figure-1 displays the association of CCI with postoperative renal function. Figure-1a demonstrates the outcome based on Model 1–a decline in GFR≥25%. The majority of patients with CCI<2 did not experience a decline in renal function (62.4%), whereas patients who experienced the outcome had CCI>3 (55.6%) (p=0.034). A similar pattern is shown in Figure-1b, where the outcome was defined as postoperative GFR≤60mL/min/1.73m^2^. Of patients with CCI<2, 60.1% did not experience the outcome whereas 64.6% of patients with CCI>2 did (p=0.014).

CCI = Charlson Comorbidity Index; GFR = Glomerular filtration rate

**Figure 2:**
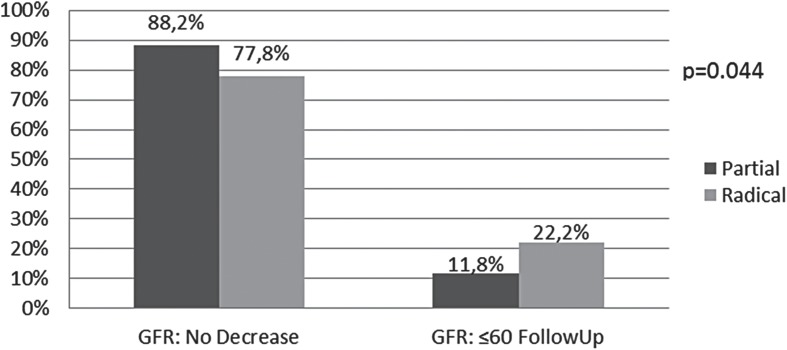
Association of Surgical Approach with Postoperative GFR, Model 2

The results of the multivariable analysis for Model 1 are displayed in Table-2. BMI (OR 1.07, 95% CI 1.02-1.11), age-adjusted CCI (OR 1.19, 95% CI 1.04-1.37), and radical nephrectomy (OR 3.09, 95% CI 1.51-6.33) were significantly associated with a decline in renal function of ≥25%. Multivariable analysis could not be conducted defining renal function as GFR≤60mL/min/1.73m^2^ due to limited data. The association of surgical approach with renal function in Model 2 is shown in Figure-2. The percentage of patients with postoperative renal function decline is nearly doubled for radical vs. partial nephrectomy (p=0.044).

GFR = Glomerular filtration rate

**Table 2:** Multivariable Analysis of Association with Decreased Renal Function-Model 1.

Variable	Odds Ratio	95% Confidence Limit
Tumor Histology	0.85	0.68-1.08
Fuhrman Grade	0.94	0.62-1.42
aCCI	1.19	1.04-1.37
Body Mass Index	1.07	1.02-1.11
Tobacco Use	0.86	0.46-1.58
Radical vs. Partial Nephrectomy	3.09	1.51-6.33
Race (Other vs. AA)	0.84	0.46-1.55
T Classification	1.06	0.86-1.30

*Significant interaction term: Fuhrman grade*T classification

## DISCUSSION

Chronic kidney disease is significantly associated with increasing morbidity, reduced quality of life, and increased mortality (18). It is also known that radical nephrectomy is associated with decreased GFR and increased risk of CKD (5). Several studies have attempted to elucidate preoperative predictors of postoperative renal decline, but a general consensus is still lacking (19–21). In the current study, we analyzed patient and tumor characteristics for their association with postoperative renal function decline. We found that increasing BMI, CCI, and radical nephrectomy were significantly associated with a postoperative decline in renal function.

BMI has recently been explored in outcomes of patients undergoing nephrectomy. Kava et al. demonstrated that lower BMI is associated with benign histopathology (HR 1.5, 95% CI 1.12-1.94) (16). The average BMI of patients with benign renal masses was 26.5 vs. 28.6 in those with malignant masses (p=0.012). When examined proportionally, among the patients with normal weight (BMI<25), one third had benign masses while only 20% of the remaining patients had benign pathology (16). In terms of cancer survival, there is conflicting data on the role of increasing BMI and mortality. In their study of almost one million US patients with various forms of cancer, Calle et al. report that increasing BMI was associated with increased cancer-specific death rates across all cancer locations examined, including kidney (22). Conversely, in a recent meta-analysis of studies examining BMI and survival in patients with RCC, Choi et al. observed that higher BMI was associated with better overall and cancer-specific survival. Patients who were either overweight or obese less often presented with symptoms and metastases compared to normal or underweight patients (23). This is similar to a previous study where increasing BMI was associated with less-favorable tumor histology and greater surgical complications yet no difference was seen in overall or cancer-specific survival (24). When examining obesity as a risk factor for RCC, it has been reported that for every 5 unit increase in BMI, the risk for RCC increases by at least 20% (25). This data contrasts with the better overall survival reported by Choi et al. and has been coined the ‘obesity paradox.’ An attempt to clarify the obesity paradox was recently reported by Hakimi et al. In their analysis of over 2000 patients who underwent surgery for a renal mass, they report that increasing BMI was associated with less advanced malignancy. Based on these findings and other studies, they hypothesize that the obesity paradox may be an observation that obese patients experience slower growing renal masses compared to their normal weight counterparts (26).

Aside from kidney cancer, renal insufficiency (GFR<60mL/min/1.73m^2^) is associated with an increased risk of death (18). It is clear that the relationship between BMI, RCC, nephrectomy, renal function, and mortality is still being defined. In the current study, we found that higher BMI is a risk factor for reduced post-operative GFR.

Our study also contributes with a novel finding in using overall comorbidity, as assessed by the CCI, as a determinant of postoperative renal decline. This adds to the previous literature indicating that diabetes and hypertension contribute to diminished renal function after nephrectomy (10, 20). Satasivam et al. proposed a method of estimating percent GFR reduction after nephrectomy based on the presence or absence of diabetes and hypertension (10). Their study examined 80 patients who underwent radical nephrectomy. The presence of diabetes and/or hypertension was examined for an association with postoperative GFR. Their patient population was relatively small, with only 7 patients having diabetes and hypertension and 41 having hypertension alone. However, they found that patients with both chronic medical conditions had a higher percent reduction in postoperative GFR compared to patients with neither diagnosis (36.4% vs. 22.8%, p<0.003). A limitation of the prior study in relation to what is addressed in the current study is the quantification of kidney function. Their study used the Modification of Diet in Renal Disease (MDRD) formula which does not take into account BMI (10). According to the US National Kidney Foundation Practice Guidelines, using a formula that accounts for gender, age, race, and BMI is superior to other methods (27). In another study assessing the association of various preoperative risk factors with postoperative renal function, Ito et al. demonstrated an independent association of hypertension with increasing creatinine (OR 4.63, 95% CI 1.75-12.23) (20). Hypertension itself leads to kidney disease in some patients, regardless of nephrectomy, so it is difficult to quantify the combination of surgery plus chronic medical conditions in their joint influence on renal function. However, it is important to note that patients with hypertension may be at greater risk for reduced GFR post-nephrectomy compared to their non-hypertensive counterparts (20).

Lastly, our study also demonstrates the association of surgical approach–namely, radical nephrectomy–with reduced renal function. Radical nephrectomy has long been an established risk factor for CKD, which spurred efforts to increase the utilization of NSS (28). Few studies have adequately controlled for preoperative renal function, and only recently data has emerged assessing the impact of surgically induced CKD in comparison to medical CKD. In their study of over 4000 patients who underwent partial or radical nephrectomy, Lane et al. compared outcomes of patients with preoperative medical CKD to patients with only surgically induced CKD. They found that patients with preoperative medical CKD had a greater rate of renal function decline (4.7% annual decline in GFR vs. 0.7%), and that overall survival was only associated with medical, not surgical, CKD (29). In their relatively short follow-up study, Khalifeh et al. found that age, CCI, and preoperative GFR are individual predictors of decreased postoperative renal function in patients receiving robotic partial nephrectomies (12). In multivariable analysis they found that only preoperative GFR was associated with latest GFR. This finding reiterates the importance of medical CKD as the predominant predictor of long-term CKD.

There are some limitations of the current study that must be taken into consideration. By nature, a retrospective analysis allows associations to be explored but cannot assess causality. In addition, our mean follow-up time is less than desired to potentially assess the true effect of a nephrectomy on long-term CKD. However, a recent prospective study noted that the postoperative decline in renal function reached a maximum by month 3, and remained stable thereafter (30). While our study does not follow trends in GFR, our follow-up time may be adequate to assess long term functionality. Strengths of our study include the detailed information collected on patient and tumor characteristics as well as the consistency seen in our results when defining the outcome in two separate methods. Additionally, through adjusting CCI for renal function we were able to capture an inclusive image of comorbidity while controlling for renal function.

In conclusion, this study demonstrates that BMI and CCI are associated with postoperative decline in renal function after nephrectomy. Additionally, radical nephrectomy is significantly associated with decreasing renal function compared to partial nephrectomy. These findings highlight the importance of clinicians assessing patient co-morbidity during the decision making process for patients presenting with a renal mass.
