# Supplementary material for: Body mass index and comorbidity are associated with postoperative renal function after nephrectomy
Source: Int Braz J Urol. 2015 Jul-Aug;41(4):697–706. doi: 10.1590/S1677-5538.IBJU.2014.0383 (PMC4756998; doi:10.1590/S1677-5538.IBJU.2014.0383)
Supplement: Related texts

# Letter: Editorial Comment

**Authors:** Francisco Fonseca

**Affiliations:** Faculdade de Medicina UNINOVE, Urology

The present study confirms, but cannot be considered definitive, that CCI and BMI negatively influence the deterioration of postoperative GFR. If they were included in the evaluation of other preoperative factors, especially those surgical associated with diabetes and hypertension, known to be related to renal damage postoperatively, BMI and CCI could have another magnitude in HR. The present work emphasizes the importance of pre–operative assessment of clinical aspects during surgical planning in order to improve the results of the final renal function. Possibly, these variables should have a different significance depending on the type of nephrectomy performed if radical or partial laparoscopic or open surgery (1). In general, patients with complex tumors are more commonly referred to open surgery, which is a known bias to their evolutionary comparison of renal function in the postoperative period (2). Annual renal functional decline was 4.7% and 0.7% for patients with chronic kidney disease due to medical causes and surgically induced chronic kidney disease, respectively, with a greater than 50% reduction in glomerular filtration rate in 7.3% and 2.2%, respectively (p<0.0001). Annual renal functional decline greater than 4.0% was associated with a 43% increase in mortality (p<0.0001) (3).

In this study, RN was performed in 67.7% of patients; this index should be made to NP, since the clinical diagnosis in stage T1a (confirmed by PT1A) was 40.9% and at PT1B in 22.7% of cases. The indication of NSS should always be evaluated for its accomplishment, as NSS decreases the chances of CKD to evolve, with the same results in relation to oncologic control (1, 2, 4, 7–9).

I believe that the authors should further investigate the real truth about the influence of BMI and CCI in the evolution of patients undergoing NSS and RN, assessing the pre and postoperative GFR because they are different populations of patients; we must evolve separately for CKD, even at different times for confirmation. As this study shows, patients undergoing NR had twice the decline of renal function (1, 5).

A fundamental parameter to evaluate the acute damage in postoperative renal function is whether there was intraoperative hypotension and/or blood transfusion, in any type of nephrectomies. Similarly, in patients undergoing partial nephrectomy is important to know which was the time of clamping of the renal artery, if the surgery was under war or cold ischemia, or if it was operated without the use of ischemia (4, 6). It is a well known complexity of the operation, directly related to the nephrometry score, and interferes with the final evolution of renal function. The type of surgery associated with the surgeon's experience has an impact on CKD.

Obese patients and with clinical comorbidities are harder to be operated and more prone to kidney failure, as well as older patients, with solitary kidney with diabetes, hypertension, and other comorbidities. Patients who have more postoperative complications are more susceptible to worsening renal function postoperatively, whether transient or definitive.

Probably the location and size of the tumor in the kidney (RENAL nephrometry score), previous renal function (GRF and/or creatinine clearance), configuration of the renal pelvis in relation to cortical, percentage of reduction of the renal parenchyma post-NSS, JRC and time of ischemia should be the most important factors for CKD patients (2, 7–10). Possibly there is an association between BMI and CCI, which should reflect directly on CKD. They may be not independent prognostic factors.

## References

[1] Go AS, Chertow GM, Fan D, McCulloch CE, Hsu CY (2004). Chronic kidney disease and the risks of death, cardiovascular events, and hospitalization. N Engl J Med.

[2] Sorbellini M, Kattan MW, Snyder ME, Hakimi AA, Sarasohn DM, Russo P (2006). Prognostic nomogram for renal insufficiency after radical or partial nephrectomy. J Urol.

[3] Lane BR, Campbell SC, Demirjian S, Fergany AF (2013). Surgically induced chronic kidney disease may be associated with a lower risk of progression and mortality than medical chronic kidney disease. J Urol.

[4] Derweesh IH, Novick AC (2005). Mechanisms of renal ischaemic injury and their clinical impact. BJU Int.

[5] Russo P (2008). Functional preservation in patients with renal cortical tumors: the rationale for partial nephrectomy. Curr Urol Rep.

[6] Zini L, Patard JJ, Capitanio U, Mejean A, Villers A, de La Taille A, Ficarra V, Crepel M, Bertini R, Salomon L, Verhoest G, Perrotte P, Bensalah K, Arjane P, Biserte J, Montorsi F, Karakiewicz P (2009). The use of partial nephrectomy in European tertiary care centers. Eur J Surg Oncol.

[7] Thompson RH, Kaag M, Vickers A, Kundu S, Bernstein M, Lowrance W (2009). Contemporary use of partial nephrectomy at a tertiary care center in the United States. J Urol.

[8] Huang WC, Levey AS, Serio AM, Snyder M, Vickers AJ, Raj GV (2006). Chronic kidney disease after nephrectomy in patients with renal cortical tumours: a retrospective cohort study. Lancet Oncol.

[9] Lane BR, Babineau DC, Poggio ED, Weight CJ, Larson BT, Gill IS (2008). Factors predicting renal functional outcome after partial nephrectomy. J Urol.

[10] Song C, Bang JK, Park HK, Ahn H (2009). Factors influencing renal function reduction after partial nephrectomy. J Urol.
